# Supplementary material for: Development of chemokine network inhibitors using combinatorial saturation mutagenesis
Source: Commun Biol. 2025 Apr 3;8:549. doi: 10.1038/s42003-025-07778-6 (PMC11969024; doi:10.1038/s42003-025-07778-6)
Supplement: Supplementary file 4 — Supplementary Data 2 [file 42003_2025_7778_MOESM4_ESM.zip › SupplementaryData3/scripts/xattune_ic50.tar!/./script/analysis/JK/JK_R25_E63_RA_HD2CM304_ATC_plot.pdf]

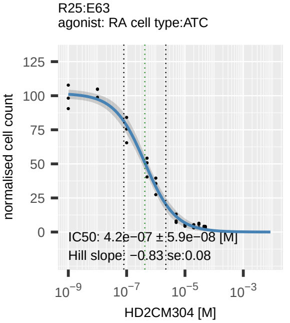
R25:E63
agonist: RA cell type:ATC
normalised cell count
125
100
75
50
25
0
IC50: 4.2e−07 ±5.9e−08 [M]
Hill slope: −0.83 se:0.08
$10^{-9}$
$10^{-7}$
$10^{-5}$
$10^{-3}$
HD2CM304 [M]
